# Antagonist Muscle Prefatigue Increases the Intracortical Communication between Contralateral Motor Cortices during Elbow Extension Contraction

**DOI:** 10.1155/2017/8121976

**Published:** 2017-07-31

**Authors:** Lejun Wang, Aidi Ma, Yuting Wang, Songhui You, Aiyun Lu

**Affiliations:** ^1^Sport and Health Research Center, Physical Education Department, Tongji University, Shanghai, China; ^2^School of Kinesiology, Shanghai University of Sport, Shanghai, China

## Abstract

To investigate the cortico-cortical coupling changes related to antagonist muscle prefatigue, we recorded EEG at FC3, C3, FC4, and C4 electrodes of twelve young male volunteers during a 30-second-long, nonfatiguing isometric elbow extension contraction with a target force level of 20% MVC before and after a sustained fatiguing elbow flexion contraction until task failure. EEG-EEG phase synchronization indices in alpha and beta frequency bands were calculated for the pre- and postfatigue elbow extension contractions. The phase synchronization index in the beta frequency band was found significantly increased between EEG of FC3-C3. The increased phase synchronization index may reflect an enhanced intracortical communication or integration of the signals between contralateral motor cortices with antagonist muscle prefatigue, which may be related to the central modulation so as to compensate for the antagonist muscle prefatigue-induced joint instability.

## 1. Introduction

Exercise-induced muscle fatigue is defined as a reversible reduction in the neuromuscular system's capacity to generate force or power [1]. It represents a complex phenomenon and encompasses a number of changes occurring at both the central and peripheral levels [2, 3]. Studies have revealed that during muscle fatigue, the activities of agonistic and antagonistic muscles are interrelated and can change in parallel with one another. Particularly, it has been interestingly found that the prefatigue of antagonist muscle may have significant influence on muscle activities, joint mechanical performance, and central voluntary activation [4, 5]. As previous researches have demonstrated the importance of the role of central mechanisms for the regulation of antagonistic muscle coactivation activities, the influence of antagonist muscle prefatigue may be closely related to the modulation of supraspinal mechanisms. However, the underlying neuromuscular control mechanism has rarely been concerned and still remains unclear.

The synchronization of neural activity across different frequencies may play an important role in the formation of neural representations [6]. Previous researchers have found the significant role of synchronization in the organization of distributed cortical activities, and the cortical control of movements involves complex facilitatory and inhibitory cortical interactions [7]. Phase synchronization analysis has been demonstrated to be a useful method to infer functional connectivity with multichannel neural signals, for example, electroencephalography (EEG) [8]. Particularly, based on EEG-EEG phase synchronization analysis method, a fatigue-induced increase in intracortical communication during cycling exercise has been found [9]. However, there is relatively little evidence that cortico-cortical coupling changes as antagonist muscle fatigued. Such evidence would provide further support for a role of synchronization across cortical regions in the organization of movement related to antagonist muscle prefatigue.

To this end, the present study aimed to examine changes of EEG-EEG phase synchronization index induced by antagonistic muscle prefatigue and thus to explore the effects of antagonist fatigue on cortico-cortical coupling and central modulation. EEG signals were recorded, and the effect of antagonist prefatigue on functional cortico-cortical coupling was determined by comparing EEG-EEG phase synchronization index during isometric elbow flexion contraction before and immediately after antagonist muscle fatigue.

## 2. Materials and Methods

### 2.1. Participants

Twelve right-handed young male volunteers (age 23.75 ± 2.49 years, height 173.08 ± 5.37 cm, and weight 64.79 ± 7.53 kg) participated in this study, which was approved by the Ethics Committee of Shanghai University of Sport. The subjects were all healthy, with no known neuromuscular disorders or musculoskeletal injuries of the neuromuscular system.

### 2.2. Experimental Protocol

The maximal elbow flexion and voluntary extension contraction torques were determined for each subject using maximum voluntary isometric elbow flexion and extension contraction (MVC) tests. Then, each subject was instructed to perform a 30-second-long, nonfatiguing isometric elbow extension contraction with the target force level at 20% MVC (prefatigue elbow extension contraction). After a sufficient rest period of 2 minutes, the subjects performed a sustained elbow-flexion-fatiguing contraction at 20% MVC until task failure (fatiguing elbow flexion contraction). As soon as the fatiguing elbow flexion contraction task was complete, the subjects were instructed to perform another 30-second-long, nonfatiguing isometric elbow extension with the target force level at 20% MVC (postfatigue elbow extension contraction).

In the experiment, the subjects sat with the upper arm vertically placed and the elbow angle kept at 90°. The right forearm was positioned parallel to the ground and supinated. When performing isometric elbow extension contraction task, the subjects were told to maintain the above posture while lifting a suitable weight from the distal part of the right forearm by means of a rope and pulley to produce a target force of 20% maximal elbow extension force. During the isometric elbow-flexion-fatiguing contraction task, a weight was suspended from the distal part of the right forearm to produce a target force of 20% maximal elbow flexion force, and the participants were told to maintain the posture by flexing the elbow with the elbow joint maintained at 90° until they felt exhausted and were no longer able to continue the contraction. The arm position was monitored by visual inspection, and feedback was given to the subjects by the same investigators for all experiments. The failure criteria for the position task were either an inability to maintain the elbow angle within 12° of the target for 5 s or displacement of the forearm from the neutral position for 5 s without correction [10]. The participants were verbally strongly encouraged to continue the sustained contraction for as long as possible. In the experiment, a self-made apparatus was used that could convert quickly between the elbow flexion and extension contraction tasks.

During the experiment, EEG signals were recorded. To reduce EEG artefacts, the experiment was conducted in a quiet, electrically shielded, dimly lit laboratory with a constant indoor temperature of approximately 24°C. The subjects were told to relax before the experiment and to gaze at a target point approximately 3 metres in front of them during the experiment session.

### 2.3. EEG Data Acquisition and Preprocessing

Using the international 10–20 electrode placement system, EEG recorded from both right and left motor cortex areas (C3, FC3, C4, and FC4) from the scalp using a 64-channel NeuroSoft SYNAMPS system (NeuroScan Labs, El Paso, TX). The scalp was cleaned with 70% ethanol before the electrode gaps were filled with conducting gel to connect the recording surface of each electrode with the scalp. The EEG data recording did not begin until the impedance for all electrodes settled below 5000*Ω*. All channels of the EEG signals were amplified (×75,000, NeuroScan SynAmps RT amplifier), band-pass filtered (0.01~100 Hz), digitized (2000 samples/s), and acquired using the NeuroScan system (NeuroScan Labs, El Paso, TX). The subjects were required to concentrate on the task performance and minimize distractions as much as possible. They were asked to maintain a stable body position and avoid eye blinks, teeth biting, and head movements during the pre- and postfatigue 30-second isometric elbow extension contraction. Possible sources of distraction or noise, such as sound or light, were minimized. During offline preprocessing, EEG signals were re-referenced to the average value of the bilateral mastoids (M1 and M2), ocular artefacts were reduced, band pass filtering at 3~60 Hz was performed using an FIR zero-phase-shift filter, artefacts were rejected (based on the criteria of signals exceeding ±100 *μ*V at any time point), and the results were visually inspected. Using the above procedure, data with apparent signal artefacts were excluded. Based on the above procedure, artefact-free 24.576-second length signals recorded during both pre- and postfatigue elbow extension contractions were acquired for each subject for later analysis.

### 2.4. EEG Power Spectrum Analysis and Nonlinear Analysis

EEG power was computed and averaged across the appropriate frequencies to obtain the power values for alpha (8–12 Hz) and beta (15–35 Hz). All power estimates were subjected to a log transformation prior to analysis, to achieve the assumption of normality [11]. Nonlinear indices including fractal dimension and sample entropy of EEG were calculated. Fractal dimension was calculated with the box-counting method as previously reported [12] while sample entropy was calculated as reported by Richman and Moorman [13]. Besides, in order to observe and confirm that the antagonist muscle do fatigue, RMS and MF of BB and TB muscles were also calculated during fatiguing elbow flexion contraction (antagonist prefatigue-induced process).

### 2.5. Phase Synchronization Analysis

EEG signals recorded during pre- and postfatigue elbow extension contractions were filtered for the frequency ranges 8–12 Hz (alpha band) and 15–35 Hz (beta band) using a 4th-order zero-phase-shift Butterworth filter. Phase synchronization analysis was then conducted, and the phase synchronization index of FC3-C3, FC4-C4, C3-C4, and FC3-FC4 during pre- and postfatigue elbow extension contractions were calculated as [14]

(1) $$\begin{array}{c} Phase\;synchronization\;index=\sqrt{{\langle \cos\theta_{xy}^{H}\left(t\right)\rangle }_{t}^{2}+{\langle \sin\theta_{xy}^{H}\left(t\right)\rangle }_{t}^{2}}, \end{array}$$

where 〈.〉_*t*_ means the average of all the values, and

(2) $$\begin{array}{c} \theta_{xy}^{H}\left(t\right)=n\theta_{x}^{H}\left(t\right)-m\theta_{y}^{H}\left(t\right), \end{array}$$

in which *θ*_*x*_^*H*^(*t*) is the phase angle calculated based on the Hilbert transformation of the EMG signals and *θ*_*y*_^*H*^(*t*) is calculated based on the EEG signals. In all cases, *m* and *n* were assigned a value of 1 according to relevant studies [14, 15].

Data processing was performed using MATLAB R2009a software (The MathWorks Inc., Natick, MA, USA).

### 2.6. Statistical Analysis

The statistical analysis was performed using SPSS 13.0 for Windows (SPSS, Inc., Chicago, IL, USA). Normality was tested using the Kolmogorov-Smirnov test. Phase synchronization index in the alpha (8~12 Hz) and beta (15~35 Hz) frequency bands, as well as power, fractal dimension, and sample entropy during pre- and postfatigue contractions were tested using paired sample *t*-tests. All significance thresholds were fixed at *α* = 0.05.

## 3. Results

**Figure 1:**
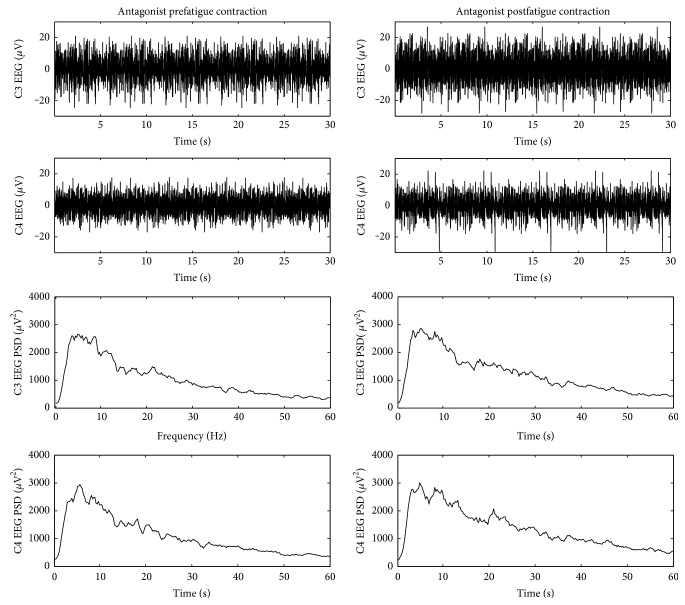
Typical examples of raw EEG signals and power spectral density function (PSD) for the EEG during pre- and postfatigue elbow extension contractions.

The fatiguing elbow flexion contractions lasted for an average of 400.2 ± 79.9 s (ranging 319~561 s). Examples of the raw EEG signals and power spectral density functions (PSDs) for the EEG are shown in Figure 1. It can be observed from the figure that during the antagonistic muscle postfatigue elbow extension contraction, EEG amplitude and power spectra of both C3 and C4 increased in 0–60 Hz compared with those during the prefatigue contraction.

**Figure 2:**
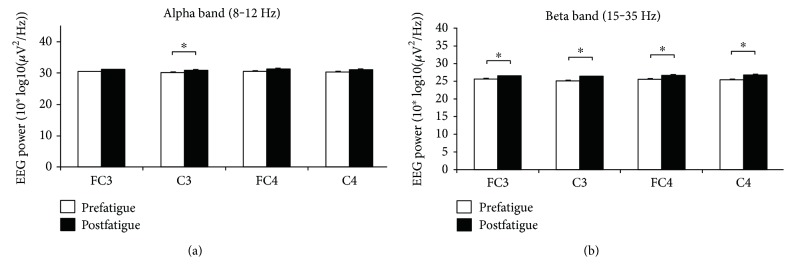
Average EEG power during pre- and postfatigue elbow extension contractions. The EEG power at C3 in the alpha band and FC3, C3, FC4, and C4 in the beta band was significantly increased during postfatigue contraction compared with that during prefatigue contraction. Data are mean ± SE. Significant differences are indicated by asterisks (*P* < 0.05).

Figure 2 shows the average EEG power in alpha and beta bands during pre- and postfatigue elbow extension contractions. The EEG power at C3 in the alpha band and FC3, C3, FC4, and C4 in the beta band was significantly increased during postfatigue contraction compared with that during prefatigue contraction (alpha band: C3: *P* = 0.009; beta bands: FC3: *P* = 0.045; C3: *P* = 0.013; FC4: *P* = 0.013; and C4: *P* = 0.013).

**Figure 3:**
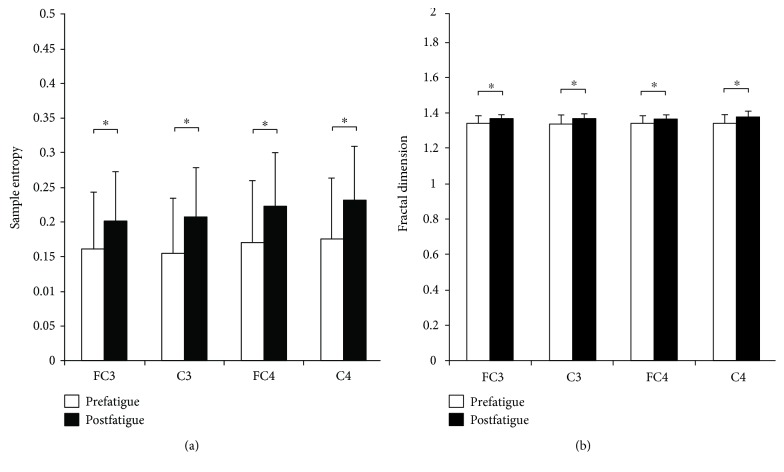
Sample entropy and fractal dimension of EEG in pre- and postfatigue elbow extension contractions. Sample entropy and fractal dimension of EEG at FC3, C3, FC4, and C4 were all found significantly increased in postfatigue contraction than in prefatigue contraction. Data are mean ± SE. Significant differences are indicated by asterisks (*P* < 0.05).

Figure 3 showed sample entropy and fractal dimension of EEG in pre- and postfatigue elbow extension contractions. Paired sample *t*-test results have revealed that sample entropy and fractal dimension of the EEG at FC3, C3, FC4, and C4 were all significantly increased during postfatigue contraction compared with those during prefatigue contraction (sample entropy: C3: *P* = 0.017, FC3: *P* = 0.040, C4: *P* = 0.037, and C4: *P* = 0.013; Fractal dimensions: C3: *P* = 0.014, FC3: *P* = 0.032, C4: *P* = 0.011, and C4: *P* = 0.006).

**Figure 4:**
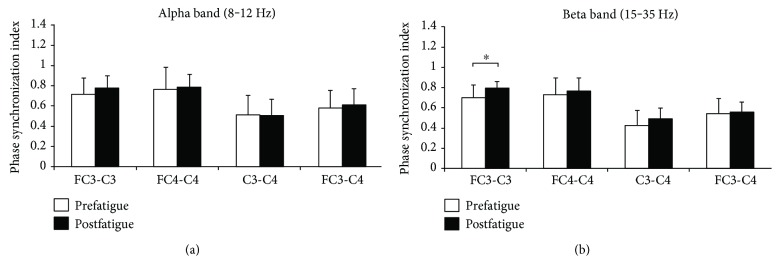
Comparisons of the phase synchronization index between EEG-EEG signals during pre- and postfatigue elbow extension contractions. The phase synchronization index in the beta frequency band between EEG of FC3-C3 electrodes was significantly increased during postfatigue elbow extension contraction compared with that during prefatigue contraction. Data are mean ± SE. Significant differences are indicated by asterisks (*P* < 0.05).

Figure 4 represents the EEG-EEG phase synchronization index during pre- and postfatigue elbow extension contractions. A paired sample *t*-test showed that the phase synchronization index in the beta frequency band between EEG of FC3-C3 electrodes was significantly increased during postfatigue elbow extension contraction compared with that during prefatigue contraction (*P* = 0.015).

## 4. Discussion

The main finding of this study is that EEG-EEG phase synchronization index in beta frequency band between contralateral primary motor cortex increased when the antagonistic muscle was prefatigued during isometric elbow extension contraction. To our knowledge, this is the first report to examine the effect of antagonistic muscle prefatigue on cortico-cortical coupling and the neural control mechanism.

It may be argued that the increase of phase synchronization index in this study may be related to the increase of EEG power in the relevant frequency band. However, no significant correlation between EEG-EEG phase synchronization index and EEG power has been discovered in previous researches [9, 16]. Besides, EEG power at FC3, C3, FC4, and C4 increased in the beta frequency band in this study while only phase synchronization index of EEG-EEG at C3 and FC3 has been found increased in the beta frequency band. All these results demonstrated that the increase of phase synchronization index cannot be explained by the enhancement of power in the corresponding frequency band.

In this study, EEG power in the beta frequency band as well as EEG sample entropy and fractal dimension of both left and right motor cortices shows a significant increase in postfatigue contraction than in prefatigue contraction. As movements of the right side of the body are mainly controlled by the left motor cortex of the brain and bilateral connection of upper limb movement [17, 18], the increase of EEG power, sample entropy, and fractal dimension in both left and right motor cortices can be easily expected. However, significant phase synchronization index changes were only found between C3 and FC3, indicating that elbow flexion muscle fatigue may only have significant influences on the functional connectivity of the contralateral motor cortex during elbow extension contraction.

EEG oscillation activity in beta rhythm is associated with motor cortical function [17, 19, 20], and a significant increase of EEG-EMG and EMG-EMG coupling in the beta frequency band induced by muscle fatigue has been revealed in many studies [15, 21, 22]. In this study, a significant increase of the phase synchronization index was found in the beta frequency band between contralateral primary motor cortices, which suggested a significant role for synchronization in the organization and regulation of elbow extension movement within the contralateral primary motor cortex when the antagonist muscle prefatigued.

It has been suggested that the central nervous system may control muscles around a joint acting synergistically as a functional unit [23]. Particularly, agonist and antagonist muscles seem to cooperate with each other as a task group in which the main task of the agonist muscle is to produce force or power while the antagonist muscle is to maintain joint stability [15]. As a result of elbow flexor prefatigue, a series of changes in peripheral and central sites related to elbow flexor and extensor muscles happens, which may influence the joint stability of motor task during the later elbow extension contraction [24]. Therefore, the increase of the phase synchronization index between contralateral motor cortices may be related to the modulation of the central nervous system so as to compensate for the antagonistic muscle prefatigue-induced joint instability, although the extent to which the intracortical physiological relationship between the researched motor areas during elbow extension task is still not fully understood.

In conclusion, phase synchronization indices in the beta frequency band were found significantly increased between contralateral motor cortices. The increased EEG-EEG phase synchronization index may reflect an enhanced intracortical communication or integration of the signals between contralateral motor cortices with antagonist muscle prefatigue, which may be related to the central modulation so as to compensate for the antagonistic muscle prefatigue-induced joint instability.
